# Feasibility and safety of endoscopic retrograde appendiceal foreign body removal in children

**DOI:** 10.3389/fped.2026.1826300

**Published:** 2026-05-07

**Authors:** Tianjiao Gao, Kuku Ge, Lina Sun, Bianhua Liu, Dan Nan, Zhao Yang, Ying Fang, Xiaoxia Ren

**Affiliations:** Department of Gastroenterology, Xi'an Children's Hospital, Xi'an, Shaanxi, China

**Keywords:** appendiceal foreign body, appendiceal preservation, children, endoscopic retrograde appendiceal therapy, foreign body removal, minimally invasive

## Abstract

**Objective:**

Endoscopic retrograde appendicitis therapy (ERAT), developed for acute appendicitis, enables minimally invasive access to the appendix for diagnosis and therapy. Based on the ERAT technique, endoscopic retrograde appendiceal foreign body removal has emerged as a logical yet technically advanced extension, offering a potential minimally invasive alternative to surgical appendectomy. The aim of this study was to evaluate the efficacy and safety of endoscopic retrograde appendiceal foreign body removal in children with appendiceal foreign bodies.

**Methods:**

This retrospective case series included six children (*n* = 6) who underwent endoscopic retrograde appendiceal foreign body removal at Xi'an Children's Hospital between January 2022 and May 2025. Clinical data, imaging findings, and procedural details were analyzed.

**Results:**

Six male patients (9.2 ± 2.2 years) were included. One patient had a clear history of foreign body ingestion but was asymptomatic. Five patients denied foreign body ingestion history and presented with varying degrees of right lower quadrant or diffuse abdominal pain. All procedures were successful without perioperative complications. Mean procedure duration was 77.5 ± 41.9 min. Retrieved foreign bodies included a wooden strip, button battery, fruit peel, metal object, fruit shell, and pinworms. Average postoperative hospitalization was 3.2 ± 1.5 days. No patient experienced recurrence of symptoms, appendicitis, or complications during the 6-month follow-up.

**Conclusion:**

Endoscopic retrograde appendiceal foreign body removal appeared feasible in this small series for removing non-sharp appendiceal foreign bodies with low-grade impaction in children, preserving appendiceal function and avoiding surgery.

## 1 Introduction

Appendiceal foreign bodies impaction is rare (incidence ∼0.005%), but can cause appendicitis, abscess, perforation, or peritonitis (1–3). In pediatric patients, appendiceal foreign bodies may be asymptomatic but carry the potential for severe complications. Common types include coins, pins, nails, screws, fishhooks, lead pellets, teeth, stones, and hair (4). Once a foreign body enters the appendiceal lumen, the presence of Gerlach's valve and insufficient peristaltic force prevent its expulsion, leading to obstruction, increased intraluminal pressure, mucosal barrier disruption, bacterial overgrowth, and inflammation (3, 5–7). Given that an appendiceal foreign body is unlikely to return to the large intestine (8), suspected impaction warrants prompt intervention (8, 9). Traditional management has largely relied on surgical appendectomy—an effective but invasive procedure associated with surgical risks, postoperative recovery, and potential long-term effects on immune function, although the clinical significance of these effects remains under investigation (10).

In recent years, the paradigm of appendiceal management has shifted toward organ preservation, particularly with the advent of endoscopic retrograde appendicitis therapy (ERAT). Originally developed for the treatment of uncomplicated acute appendicitis, ERAT enables direct intraluminal access to the appendix via a colonoscopic approach, allowing for diagnostic visualization, irrigation, and therapeutic intervention (11). Building upon this innovative platform, endoscopic retrograde appendiceal foreign body removal has emerged as a logical yet technically advanced extension, offering a potential minimally invasive alternative to surgical appendectomy.

To date, only a few case reports have described endoscopic removal of appendiceal foreign bodies in children. Freeman et al. recently reported successful endoscopic retrieval of high-powered magnets from the appendiceal orifice in an asymptomatic 10-year-old boy, representing the first such case performed prior to the development of appendicitis (12). Tang et al. described endoscopic removal of an appendiceal foreign body using a disposable pancreaticobiliary imaging catheter in a 13-month-old girl (2). These previous reports suggest that endoscopic removal is technically feasible in select pediatric cases, but the overall experience remains limited, and no case series has systematically evaluated the feasibility and safety of this approach across multiple pediatric patients. Children present unique anatomical and physiological challenges, including a thinner appendiceal wall, a relatively underdeveloped omentum, and a heightened risk of rapid progression to perforation and peritonitis. Thus, there is an urgent need to evaluate whether ERAT-based foreign body retrieval can be safely and effectively adapted for pediatric use.

This study aims to report our experience with endoscopic retrograde appendiceal foreign body removal in pediatric patients. We describe the technical approaches employed and evaluate procedural success, complication rates, and short-term outcomes.

## 2 Materials and methods

### 2.1 Study population

This was a retrospective case series. All children who presented to Xi'an Children's Hospital with appendiceal foreign bodies between January 2022 and May 2025 were identified through a review of our institutional endoscopy database and electronic medical records. Children (0–14 years) scheduled for endoscopic retrograde appendiceal foreign body removal at Xi'an Children's Hospital (January 2022–May 2025) were enrolled. Inclusion criteria: 1) Suspected appendiceal foreign bodies based on symptoms, signs, laboratory tests, imaging examinations, or direct visualization during ERAT; 2) Parental informed consent. Exclusion criteria: 1) Diffuse peritonitis, appendiceal abscess, appendiceal perforation, or appendiceal gangrene; 2) Contraindications to colonoscopy or anesthesia. During the study period, 7 children were diagnosed with appendiceal foreign bodies. Among them, 1 presented with appendiceal abscess, and the patient underwent urgent surgical appendectomy. The remaining 6 patients met the inclusion criteria and underwent endoscopic retrograde appendiceal foreign body removal. The study was ethically approved by the Institutional Review Board of Xi'an Children's Hospital (No. 20220102). Informed consent forms in accordance with the Declaration of Helsinki was obtained from the guardians of the children.

### 2.2 Procedure

All patients underwent standardized bowel preparation (13, 14). Under general anesthesia with endotracheal intubation.

#### 2.2.1 Endoscopic retrograde appendiceal foreign body removal assisted by biliopancreatic duct imaging system

The biliopancreatic duct imaging system (eyeMax™, 9Fr, Micro-Tech, Nanjing, China) is a disposable, catheter-based endoscopic visualization system originally designed for pancreaticobiliary duct exploration. It consists of a high-resolution imaging fiber integrated into a 9Fr catheter, allowing direct visualization of luminal structures during advancement. This system was used for appendiceal evaluation because it provides real-time, direct visualization of the appendiceal lumen and its contents, including the identification of foreign bodies, fecaliths, mucosal abnormalities (e.g., hyperemia, edema, erosion, pus), and luminal strictures. Unlike x-ray fluoroscopy, which relies on indirect contrast imaging, or ultrasound, which has limited resolution for intraluminal details, the biliopancreatic duct imaging system enables targeted irrigation and device-guided retrieval under direct vision, potentially improving procedural precision and reducing radiation exposure.

The child was placed in the left lateral decubitus or supine position. After the colonoscope with a transparent cap was advanced to the ileocecal region, the appendiceal orifice was fully exposed to observe whether there was hyperemia, edema, bleeding, or purulent discharge in the mucosa at the appendiceal orifice. The Gerlach's valve (appendiceal valve) was gently retracted using the transparent cap. The disposable biliopancreatic duct imaging catheter was slowly advanced into the appendiceal lumen through the endoscopic biopsy channel, with continuous water injection during advancement for observation. The conditions of the mucosa in the appendiceal lumen, such as hyperemia, edema, erosion, and fecaliths, were observed. If there was a foreign body in the appendiceal lumen, it was removed by saline irrigation or using appropriate devices (e.g., grasping forceps, retrieval basket, extraction balloon) under visualization. The endoscope was withdrawn after the appendiceal lumen was thoroughly.

#### 2.2.2 Endoscopic retrograde appendiceal foreign body removal assisted by x-ray fluoroscopy

The child was placed in the left lateral decubitus or supine position. After the colonoscope with a transparent cap was advanced to the ileocecal region, the appendiceal orifice was fully exposed to observe whether there was hyperemia, edema, bleeding, or purulent discharge in the mucosa at the appendiceal orifice. The Gerlach's valve (appendiceal valve) was gently retracted using the transparent cap. A guidewire was inserted into the appendiceal lumen, and sphincterotome was advanced over the guidewire for cannulation. The guidewire insertion was stopped when resistance was felt, and after slow withdrawal of the guidewire, the insertion depth was measured. The insertion depth of the sphincterotome was determined based on the guidewire insertion depth, and cannulation was performed under x-ray fluoroscopic guidance as much as possible to confirm whether the catheter had reached the appendiceal blind end. For x-ray-guided procedures, a 5 ml syringe was used to inject contrast agent under x-ray fluoroscopy for imaging of the appendiceal lumen, allowing observation of the appendix's course, morphology, luminal diameter, inner wall, obstruction, and stenosis. The appendiceal lumen was repeatedly irrigated with saline via a syringe and the colonoscope's water channel, and the intraluminal foreign body was retrieved by saline irrigation or appropriate devices (e.g., retrieval basket, extraction balloon).

Procedures were performed by two experienced endoscopists. Liquid diet was resumed 6 h post-procedure.

### 2.3 Observation indicators

Demographics, time since ingestion or symptom onset, clinical presentation, laboratory results [white blood cell count (WBC), neutrophil percentage, high sensitivity C-reactive protein (hs-CRP)], imaging findings (abdominal ultrasound, x-ray, CT), procedural details (duration, technique, complications), postoperative recovery (pain resolution, length of stay), and 6-month follow-up data were recorded.

### 2.4 Statistical analysis

Categorical data are presented as frequencies (%). Normally distributed continuous data are expressed as mean ± SD; non-normally distributed data as median (IQR). Normality was assessed using the Kolmogorov–Smirnov test.

## 3 Results

### 3.1 Baseline patient characteristics

All 6 patients were male (9.2 ± 2.2 years). One asymptomatic patient had a clear foreign bodies ingestion history. Five patients denied foreign bodies ingestion history and presented with right lower quadrant or diffuse abdominal pain. One patient had vomiting. Two patients had nausea (Table 1).

**Table 1. Clinical:** manifestations of appendiceal foreign bodies in children.

Patient	Gender	Age (Years)	Time since ingestion	Symptoms and duration	Associated symptoms	Signs
1	Male	9	Unknown	Right lower quadrant pain, 1 month	None	Right lower quadrant tenderness
2	Male	9	2 days	None	None	None
3	Male	9	Unknown	Right lower quadrant pain, 2 months	Vomiting	Right lower quadrant tenderness
4	Male	13	Unknown	Diffuse abdominal pain, 27 days	Nausea	Diffuse abdominal tenderness
5	Male	9	Unknown	Right lower quadrant pain, 4 months	Nausea	Right lower quadrant tenderness
6	Male	6	Unknown	Abdominal pain, 1 month	None	Right lower quadrant tenderness

### 3.2 Imaging and laboratory findings

Preoperative imaging (abdominal ultrasound, x-ray, or CT) confirmed or suggested appendiceal foreign bodies in five of six patients (Patients 1, 2, 4, 5, and 6). Patient 3 had negative imaging findings. Laboratory results were within normal ranges for all patients. The WBC, neutrophil percentage, and hs-CRP of the 6 children before procedure were (6.5 ± 1.3)   ×   10^9^/L, 53.3 (39.6, 55.6)%, and 0.55 (0.15, 2.30) mg/L, respectively. Detailed imaging and laboratory data are presented in Table 2.

**Table 2:** Imaging and laboratory findings in children with appendiceal foreign bodies.

Patient	Abdominal ultrasound	Abdominal x-ray (erect & lateral)	Abdominal CT	WBC (×10^9^/L)	Neutrophils (%)	Hs-CRP (mg/L)
1	Appendicitis	Not performed	Not performed	6.16	56.3	0.31
2	Hyperechoic focus (2.5 × 1.6 mm) within appendiceal lumen with comet tail artifact, suggestive of appendiceal foreign body	Round hyperdense opacity (0.8 cm diameter) in right lower quadrant	Not performed	7.74	55.3	0.78
3	Negative	Negative	Negative	8.42	54.1	5.42
4	Negative	Small nodular hyperdense opacity in right pelvis, suggestive of foreign body	Hyperdense nodular shadow (6 mm, CT value 2,139 HU) in right pelvis, suggestive of appendiceal foreign body; Small amount of pelvic fluid	6.56	52.5	1.26
5	Negative	Negative	Hyperdense shadow within appendiceal lumen	5.37	39.2	0.11
6	Appendiceal fecal residue	Not performed	Appendiceal fecalith	4.94	39.7	0.16

### 3.3 Endoscopic findings and procedure details

**Figure 1:**
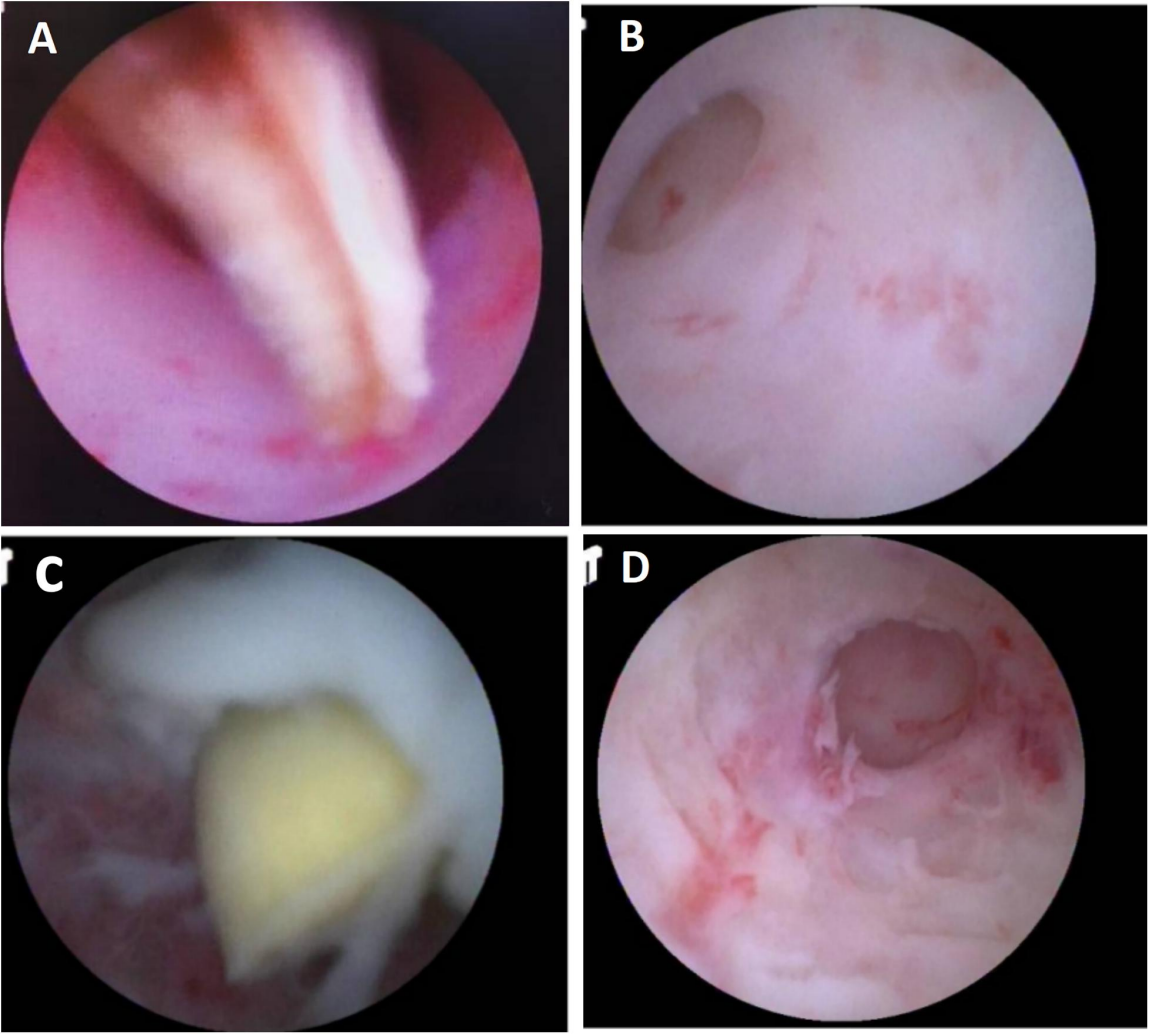
Endoscopic findings during endoscopic retrograde appendiceal foreign body removal assisted by biliopancreatic duct imaging system (patient 1). **(A)** Wooden foreign body within the appendix; **(B)** Appendiceal luminal stenosis; **(C)** Fecolith and purulent secretions in appendiceal lumen; **(D)** Appendiceal apex.

**Figure 2:**
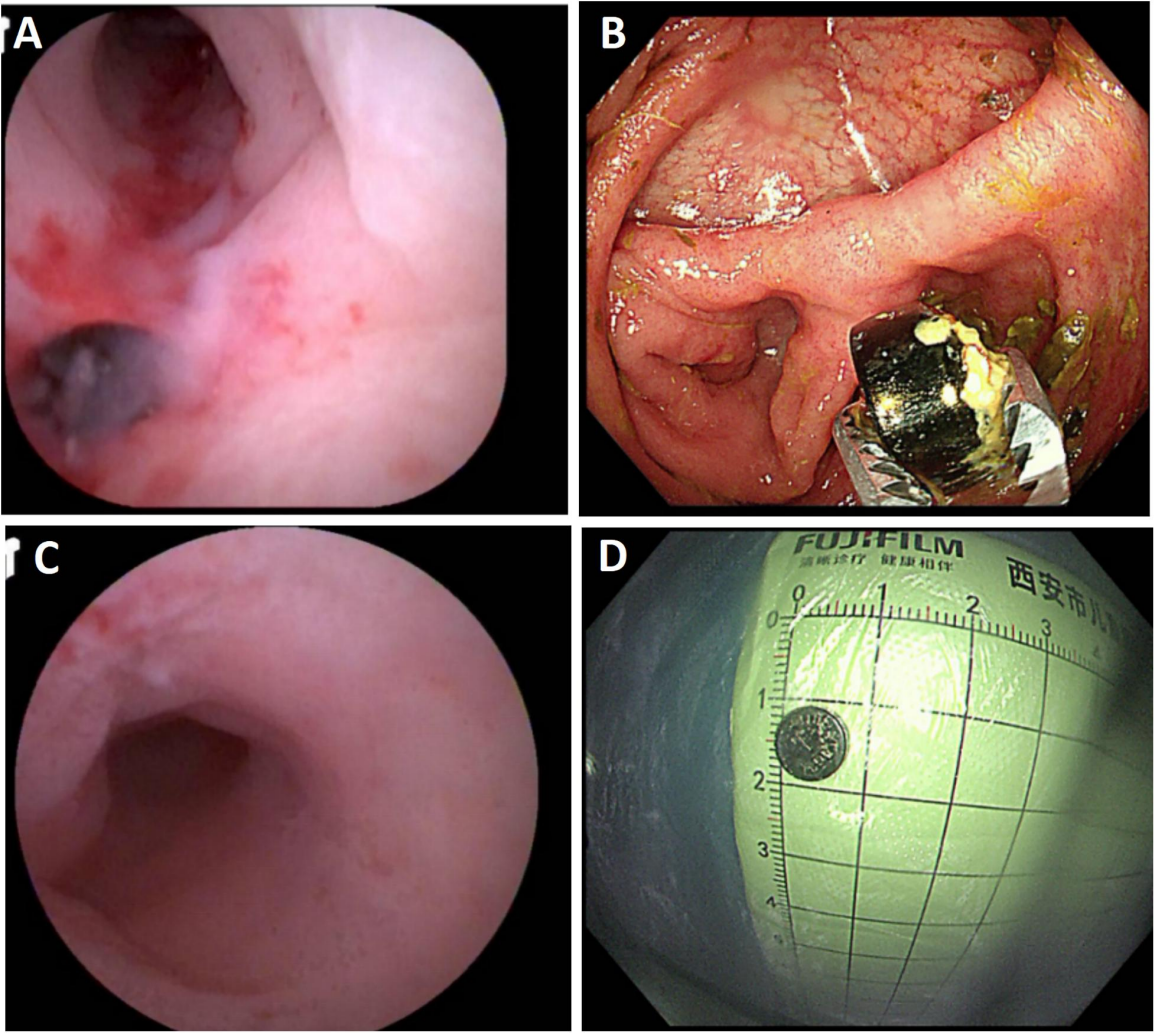
Endoscopic findings during endoscopic retrograde appendiceal foreign body removal assisted by biliopancreatic duct imaging system (patient 2). **(A)** Foreign body impacted within the appendiceal lumen; **(B)** Removed the foreign body with foreign body forceps; **(C)** Mucosal hyperemia and edema of the appendiceal wall; **(D)** Retrieved button battery.

**Figure 3:**
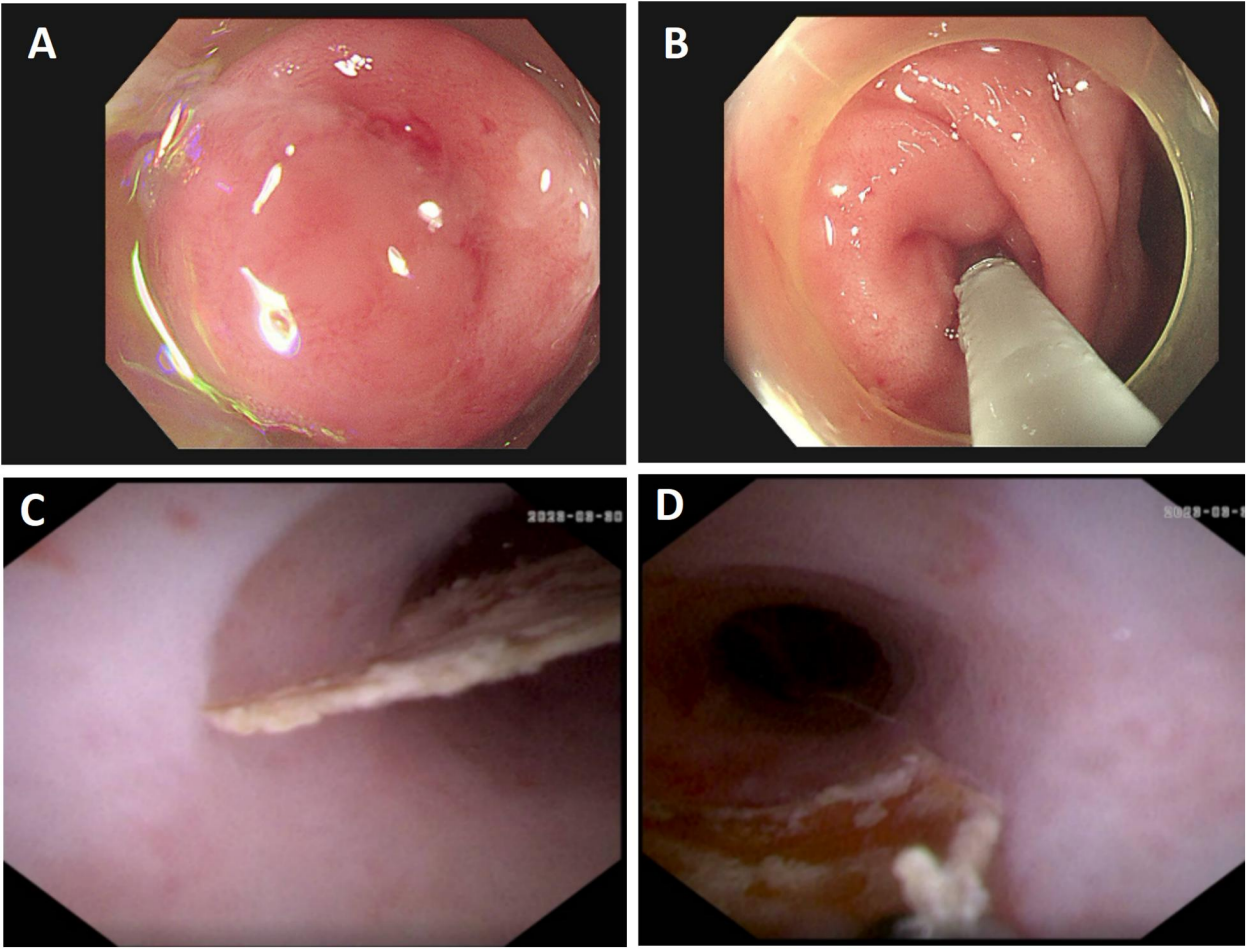
Endoscopic findings during endoscopic retrograde appendiceal foreign body removal assisted by biliopancreatic duct imaging system (patient 3). **(A)** Swelling of the appendiceal orifice; **(B)** Advancement of the biliopancreatic duct imaging catheter into the appendiceal orifice; **(C)** Foreign body within the appendiceal lumen; **(D)** Grasping and extraction of irregular fruit peel using forceps.

**Figure 4:**
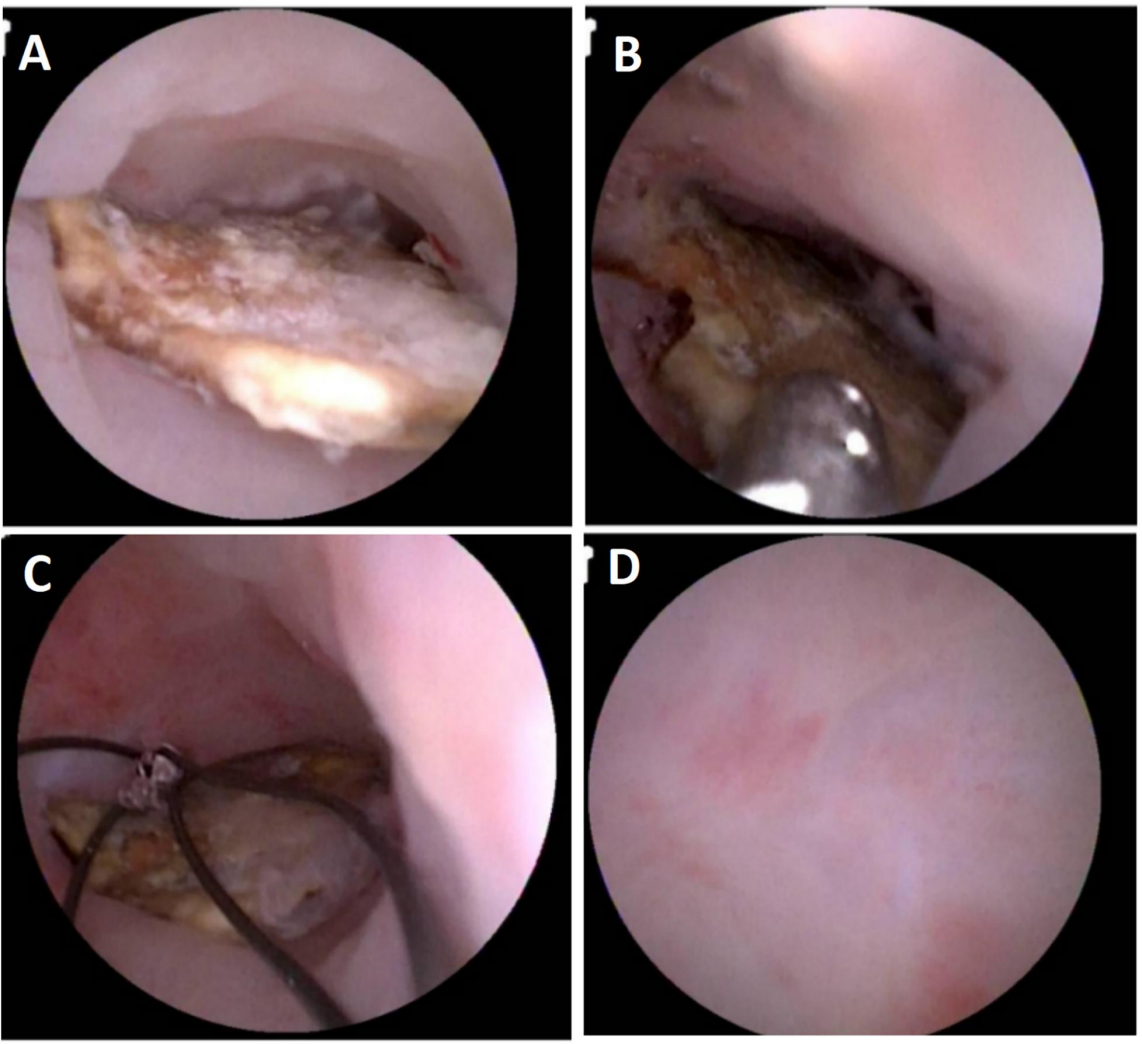
Endoscopic findings during endoscopic retrograde appendiceal foreign body removal assisted by biliopancreatic duct imaging system (patient 5). **(A)** Impacted foreign body within appendiceal lumen; **(B)** Grasping of foreign body with grasping forceps; **(C)** Capturing foreign body using retrieval basket; **(D)** Appendiceal apex with intact mucosa.

**Figure 5:**
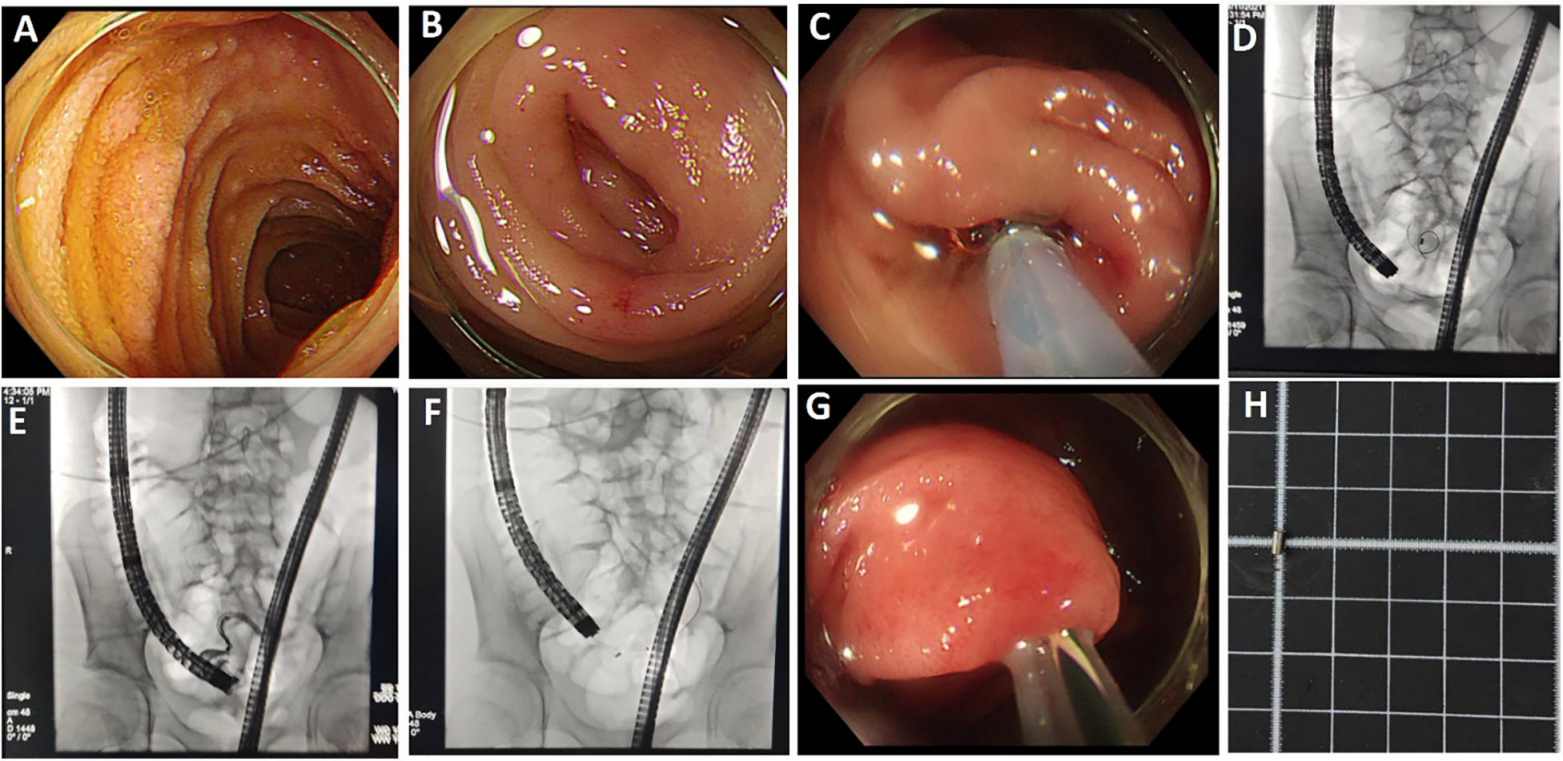
Endoscopic findings during endoscopic retrograde appendiceal foreign body removal assisted by x-ray fluoroscopy (patient 4). **(A)** Terminal ileum; **(B)** Appendiceal orifice; **(C)** Cannulation of appendiceal lumen; **(D)** Guidewire advancement into appendiceal lumen; **(E)** Fluoroscopic contrast imaging of appendiceal lumen; **(F)** Luminal clearance using an extraction balloon; **(G)** Balloon sweeping through appendiceal lumen; **(H)** Retrieved cylindrical metal foreign body.

**Figure 6:**
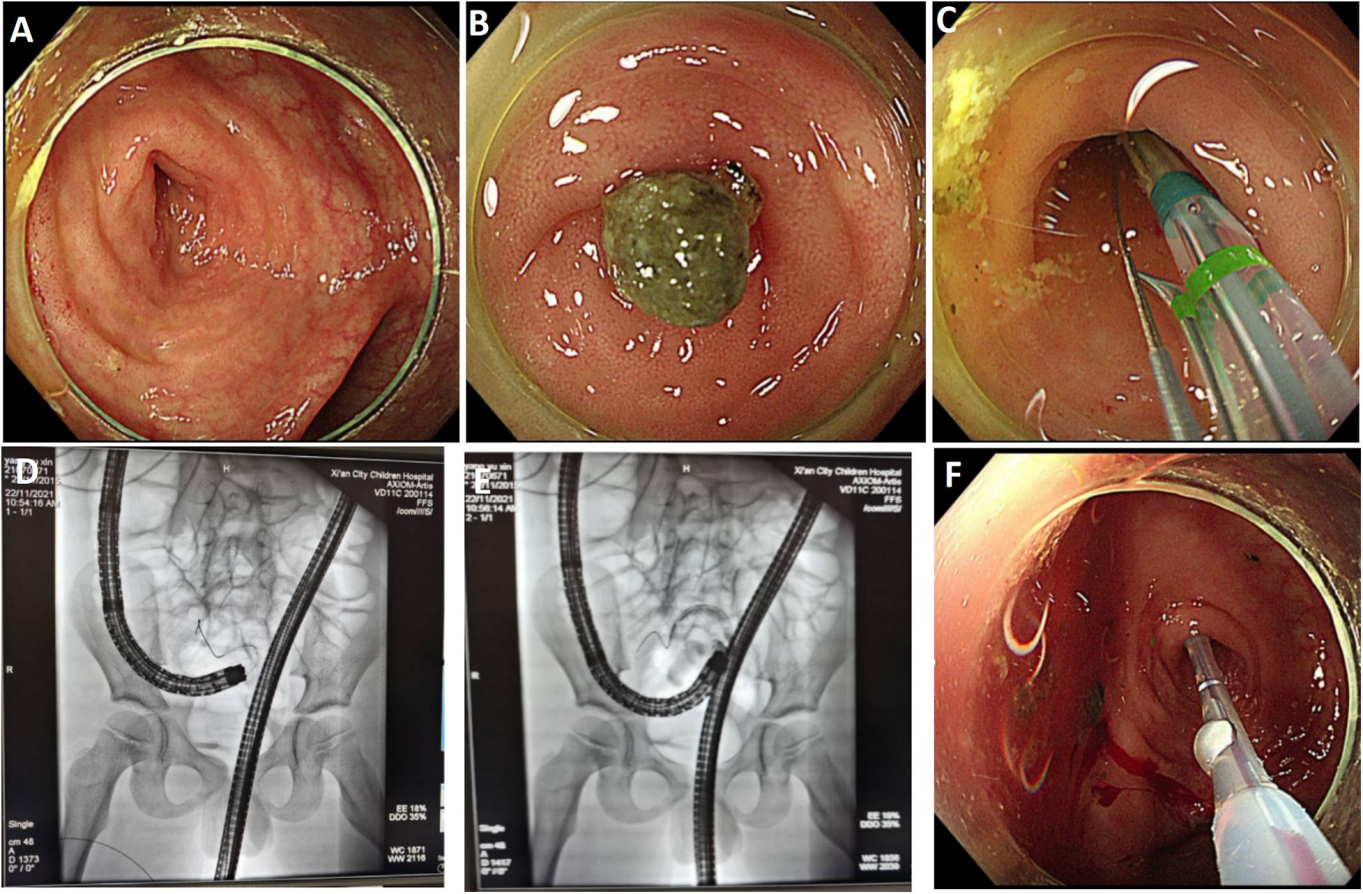
Endoscopic findings during endoscopic retrograde appendiceal foreign body removal assisted by x-ray fluoroscopy (patient 6). **(A)** Appendiceal orifice; **(B)** Appendiceal fecalith; **(C)** Appendiceal lumen cannulation; **(D)** Guidewire advancement into the appendiceal lumen; **(E)** Appendiceal lumen angiography; **(F)** Numerous pinworms.

All six procedures were technically successful. The biliopancreatic duct imaging system was used in Patients 1 (Figure 1, Supplementary Video 1), 2 (Figure 2), 3 (Figure 3), and 5 (Figure 4) (4 cases), and x-ray fluoroscopy was used in Patients 4 (Figure 5) and 6 (Figure 6) (2 cases). Retrieved foreign bodies included a wooden strip, button battery, fruit peel, cylindrical metal object, fruit shell, and pinworms (Table 3).

**Table 3:** Endoscopic findings and treatment outcomes of endoscopic retrograde appendiceal foreign body removal in children.

Patient	Endoscopic appendiceal findings	Foreign body type	Retrieval method	Procedure time (min)	Complications	Time to pain resolution (days)	Hospital stay (days)
1	Luminal stenosis; Mucosal hyperemia, edema, and erosion; Fecoliths and purulent secretions	Strip-shaped wooden object (1.0 cm length)	Saline irrigation	80	None	1	4
2	Mucosal hyperemia and edema	Button battery (0.8 cm diameter)	Saline irrigation + forceps	45	None	N/A	1
3	Swelling of appendiceal orifice	Irregular fruit peel	Forceps	70	None	2	5
4	Normal appendiceal orifice	Cylindrical metal object (0.5 cm diameter)	Extraction balloon	90	None	1	3
5	Smooth and intact mucosa	Irregular hard fruit shell	Combined forceps and basket	150	None	1	2
6	Fecalith obstructing the appendiceal orifice	Pinworms	Saline irrigation	30	None	1	4

All 6 patients underwent successful endoscopic retrograde appendiceal foreign body removal. Mean procedure duration was 77.5 ± 41.9 min. No perioperative complications occurred. Postoperative antibiotics were administered to Patient 1 (7 days), who had confirmed acute appendicitis. Patient 6 received albendazole for pinworm infection. No other patients required post-procedure antibiotics or additional interventions. Average postoperative hospitalization was 3.2 ± 1.5 days (Table 3).

### 3.4 Follow-up

No patient experienced fever, abdominal pain, nausea, vomiting, or recurrent appendicitis during the 6-month follow-up.

## 4 Discussion

In this retrospective case series of six pediatric patients, endoscopic retrograde appendiceal foreign body removal was successfully performed without complications, and no symptom recurrence or appendicitis was observed during 6-month follow-up. These findings suggest that this ERAT-based technique may offer a feasible, safe, and organ-preserving alternative to surgical appendectomy for selected children with non-sharp, low-grade impacted appendiceal foreign bodies.

Diagnosing appendiceal foreign bodies in children requires comprehensive consideration of clinical symptoms, foreign body type and size, and time since ingestion. A physical examination should also be performed to assess for signs of peritonitis. The absence of obvious symptoms or signs in some children significantly increases diagnostic difficulty. The most common imaging modalities are abdominal ultrasound, x-ray, and CT; diagnostic laparoscopy is occasionally used. CT scan can identify the foreign body's location and size and detect complications like appendicitis secondary to the foreign body (15). Typically, appendicitis is diagnosable on CT scan by findings such as appendiceal enlargement, wall enhancement and thickening, and peri-appendiceal fat stranding or inflammatory changes. However, atypical imaging findings in some children make the diagnosis challenging. The pediatric appendix is longer and thinner than the adult appendix, allowing for earlier perforation. Due to the shorter omentum, which cannot adequately cover a perforation site before age 10, children are prone to generalized peritonitis. Consequently, the diagnosis of appendicitis in children is often relatively delayed, carrying a much higher risk of complications compared to adults.

The appendix is an important immune organ and a key component of the intestinal immune barrier (16). It also exerts a protective effect against colorectal cancer (17). Therefore, the concept of preserving the appendix while treating appendiceal pathology is gaining wider recognition among professionals. This has led to the development of ERAT. Although ERAT has been increasingly applied in the management of acute appendicitis, its utility may extend to other appendiceal conditions requiring intraluminal intervention, such as chronic abdominal pain secondary to appendiceal foreign bodies or fecaliths. Based on the ERAT technique, endoscopic retrograde appendiceal foreign body removal has become a possibility. Compared to surgery, endoscopic retrograde appendiceal foreign body removal enables the “ultra-minimally invasive” endoscopic removal of the foreign body, relieving appendiceal obstruction. This approach rapidly alleviates symptoms, preserves the appendix, and offers reduced trauma, lower risk, and faster recovery – aligning better with the needs of both children and parents. This study summarizes the therapeutic role of endoscopic retrograde appendiceal foreign body removal in children with appendiceal foreign bodies.

Patient 1 had appendicitis caused by a long, wooden foreign body impacted in the appendiceal lumen. Ultrasound showed a thickened, swollen appendiceal tip (external diameter 4.8–7.6 mm), intraluminal fluid, and slight swelling with increased echogenicity in the surrounding mesentery. Direct visualization via disposable biliopancreatic duct imaging catheter revealed congested and edematous mucosa with white flocculent material and luminal narrowing in the mid-to-distal appendix. Patients 2 and 4 had preoperative imaging confirming appendiceal foreign bodies unlikely to pass spontaneously. Patient 3 presented with 2 months of right lower quadrant pain and right lower quadrant tenderness on exam. Colonoscopy showed swelling at the appendiceal orifice; the disposable biliopancreatic duct imaging catheter visualized a difficult-to-pass irregular fruit peel retrieved successfully with forceps, resolving the pain within 2 days post-procedure. Patient 5 had a preoperative abdominal CT showing a nodular high-density shadow in the appendiceal lumen. The disposable biliopancreatic duct imaging catheter revealed an impacted, irregular, hard fruit pit. Repeated attempts with a retrieval basket were unsuccessful; switching to grasping forceps allowed grasping and moving the foreign body proximally, after which a basket successfully retrieved it upon slow scope withdrawal. Patient 6, preoperative abdominal ultrasound and CT suggested an appendiceal fecalith. During the operation, fecalith obstruction was found at the appendiceal orifice. The appendiceal fecalith was dislodged using a disposable sphincterotome, and after repeated irrigation of the appendiceal lumen with saline, yellowish concretion-like stones and numerous pinworms were observed to be expelled.

So far, the literature on endoscopic retrograde appendiceal foreign body removal in children has been limited to case reports. For instance, Tang et al. demonstrated the feasibility of direct visualization-guided retrieval using a disposable pancreaticobiliary imaging catheter in a 13-month-old girl, while Freeman et al. reported the first pre-appendicitis endoscopic removal of high-powered magnets from the appendiceal orifice in an asymptomatic 10-year-old boy (2, 12). However, these reports were limited to single cases, and none systematically evaluated the ERAT-based approach across a series of pediatric patients. In comparison, our study represents the case series (*n* = 6) of endoscopic retrograde appendiceal foreign body removal in children using ERAT-based techniques. Unlike previous reports that focused on single foreign body types (e.g., magnets, pancreaticobiliary catheter removal), our series included a diverse range of foreign bodies (wooden strip, button battery, fruit peel, metal object, fruit shell, and pinworms). Furthermore, while prior studies described either x-ray fluoroscopy or direct visualization alone, our series employed both guidance modalities (biliopancreatic duct imaging system in 4 patients, x-ray fluoroscopy in 2 patients), demonstrating the versatility of ERAT-based approaches. Finally, all six procedures were technically successful without complications, and no recurrence was observed during 6-month follow-up, suggesting that this technique may be a safe and effective organ-preserving option in selected pediatric cases.

ERAT can be performed under ultrasound, x-ray, or biliopancreatic duct imaging system guidance. Ultrasound-guided ERAT offers advantages of convenience, lack of radiation, and lower requirements for personnel, equipment, and space. However, it provides less intuitive and clear visualization of appendiceal morphology, structure, and micro-perforations compared to x-ray. X-ray-guided ERAT is more suitable for confirming cannulation position, identifying perforations, and retrieving fecaliths. Its drawbacks include higher equipment demands, complex operation, and risks of contrast allergy, residual contrast, and ionizing radiation. While both ultrasound and x-ray guidance allow observation of the appendiceal orifice and surrounding mucosa, and can detect appendiceal inflammation to some extent, neither can directly visualize the intraluminal contents of the appendix. This poses limitations for diagnosing early or atypical appendicitis. Biliopancreatic duct imaging system-guided ERAT allows direct inspection of the appendiceal lumen under vision, enabling more accurate assessment for inflammation and providing definitive diagnostic evidence for appendicitis.

Several limitations should be noted in this study. First, the small sample size (*n* = 6) and single-center, retrospective design limit the generalizability of our findings and may introduce selection bias. Second, patient selection was highly specific: only children with non-sharp, low-grade impacted appendiceal foreign bodies and without evidence of complicated appendicitis (e.g., diffuse peritonitis, appendiceal abscess, perforation, or gangrene) were enrolled for endoscopic intervention. Third, the two different imaging guidance modalities (biliopancreatic duct imaging system vs. x-ray fluoroscopy) were used based on endoscopist preference and equipment availability, which may have introduced variability in procedural duration and technique. Fourth, the 6-month follow-up period may be insufficient to detect long-term complications or delayed recurrent appendicitis. Longer-term prospective studies with larger, multicenter cohorts are needed to further validate the safety and efficacy of this approach and to better define optimal patient selection criteria.

## 5 Conclusion

In this retrospective case series of six pediatric patients, endoscopic retrograde appendiceal foreign body removal may represent a potential minimally invasive option for removing selected (non-sharp, low-grade impaction) appendiceal foreign bodies in children. The technique preserves the appendix, aligns with ultra-minimally invasive and enhanced recovery principles, and may offer a valuable alternative to surgery in appropriately selected cases. Given the small sample size and descriptive nature of this study, further investigations with larger cohorts are warranted to establish definitive safety and efficacy.

## Data Availability

The original contributions presented in the study are included in the article/Supplementary Material, further inquiries can be directed to the corresponding author.
